# Muscle-Sparing Latissimus Dorsi Flap with a Longitudinal Muscle Cuff: A Practical Strategy for Reliable Harvest of Elongated Skin Paddles

**DOI:** 10.3390/jcm15187296

**Published:** 2026-09-19

**Authors:** Yooseok Ha, Suein Lee, Seung Eun Hong, Youn Hwan Kim

**Affiliations:** 1 Department of Plastic and Reconstructive Surgery, College of Medicine, Chungnam National University, Daejeon 35015, Republic of Korea; useok4u@naver.com; 2 Department of Plastic and Reconstructive Surgery, Hanyang University Hospital, Seoul 04763, Republic of Korea; lsieviee@gmail.com; 3 Department of Plastic and Reconstructive Surgery, School of Medicine, Ewha Womans University, Seoul 07804, Republic of Korea; 4 Department of Plastic and Reconstructive Surgery, College of Medicine, Hanyang University, Seoul 04763, Republic of Korea

**Keywords:** surgical flaps, superficial back muscles, myocutaneous flap, perforator flap, free tissue flaps, plastic surgery procedures

## Abstract

**Background/Objectives**: Reconstruction of elongated soft-tissue defects requires flaps that provide sufficient longitudinal reach without excessive width or donor-site morbidity. We describe a modified latissimus dorsi (LD) flap in which a narrow, longitudinally oriented muscle cuff is incorporated along the long axis of the skin paddle to act as a vascular carrier, and evaluate its clinical feasibility and reliability. **Methods**: This retrospective case series included consecutive patients who underwent soft-tissue reconstruction with an LD flap incorporating a longitudinal muscle component beneath an elongated skin paddle at a single tertiary center between March 2020 and August 2025. The same longitudinal muscle-cuff concept was applied across a spectrum of muscle harvest: most flaps were muscle-sparing LD (msLD; limited longitudinal muscle segment with preservation of the horizontal thoracodorsal nerve branch), while a wider cuff requiring sacrifice of both nerve branches resulted when the cuff extended proximal to the thoracodorsal bifurcation and was designated musculocutaneous LD (mcLD). Outcomes included flap survival, partial skin-paddle necrosis, operative time, donor-site closure method, and donor-site morbidity. In an exploratory subgroup analysis, the cohort was divided at a skin-paddle length-to-width ratio of 2.5 and compared using Welch’s *t*-test and Fisher’s exact test. **Results**: Twenty-five flaps were analyzed (21 msLD, 84.0%; 4 mcLD, 16.0%). The mean patient age was 54.0 ± 19.9 years, and scar contracture was the most common etiology (56.0%), with the lower leg the most common defect site (44.0%). The mean skin-paddle dimensions were 26.8 ± 3.2 × 11.4 ± 1.8 cm (mean area, 305.3 ± 63.8 cm^2^), and the mean operative time was 154.8 ± 49.7 minutes. No total flap loss occurred, and partial necrosis of limited extent developed in two patients (8.0%). Primary donor-site closure was achieved in 14 patients (56.0%). Because long-axis length was comparable between groups, the ≥2.5 group had a narrower skin paddle (10.0 ± 0.4 vs. 12.9 ± 1.5 cm) and smaller flap area (273.5 ± 43.7 vs. 339.7 ± 65.6 cm^2^) as an arithmetic consequence of the group definition; primary donor-site closure was more frequent in this group (76.9% vs. 33.3%, *p* = 0.047, unadjusted), and partial necrosis did not differ (7.7% vs. 8.3%, *p* = 1.00). No clinically significant donor-site functional complaints were documented in the medical records over a mean follow-up of 15.0 ± 8.8 months, and the mean QuickDASH score was 2.82 ± 3.10 (range, 0–11.36), although the questionnaire was administered postoperatively only and no objective strength or range-of-motion testing was performed. **Conclusions**: Harvest of long, relatively narrow LD skin paddles using a longitudinally oriented muscle cuff was feasible and was associated with favorable flap survival in this series, with no total flap loss and a low rate of limited partial necrosis. Because the design retains most of the muscle and, in the msLD configuration, its horizontal motor innervation while avoiding extensive intramuscular perforator dissection, it may represent a practical option for elongated soft-tissue defects; the absence of a control group and of objective perfusion or shoulder-function assessment precludes conclusions about mechanism or superiority.

## 1. Introduction

Reconstruction of extensive soft-tissue defects often requires a flap that provides not only a large surface area but also sufficient longitudinal reach. This requirement is particularly relevant for elongated defects of the extremities, where a long and relatively narrow skin paddle may provide efficient coverage while avoiding unnecessary flap width and donor-site burden. Although contemporary perforator flaps allow considerable flexibility in flap design, maintaining reliable perfusion throughout an increasingly large or elongated skin paddle remains an important consideration.

The anterolateral thigh (ALT) flap has become a workhorse flap for reconstruction of large soft-tissue defects because of its long vascular pedicle, large available skin territory, and versatility in tissue composition and flap design [1]. However, the vascular anatomy of the ALT flap is highly variable, particularly with regard to the origin, course, and number of perforators [1]. Large skin paddles may survive on a single dominant perforator in appropriately selected cases; nevertheless, as flap dimensions increase, perforator location, number, and interperforator vascular connections become increasingly relevant to perfusion reliability. Similar concerns regarding perfusion of remote skin territories have been demonstrated in other perforator-based flaps. In deep inferior epigastric perforator (DIEP) flaps, for example, perfusion progressively decreases in regions farther from the dominant perforator, particularly in the traditional zone IV territory [2]. These observations highlight the challenge of achieving reliable perfusion when designing unusually large or elongated perforator-based skin paddles.

The latissimus dorsi (LD) flap represents another established option for reconstruction of extensive soft-tissue defects because of its consistent vascular anatomy, large available skin territory, and reliable blood supply. Conventional musculocutaneous LD flaps can provide substantial amounts of well-vascularized tissue but require sacrifice of a considerable portion of the muscle, with the potential for postoperative shoulder dysfunction and donor-site morbidity [3]. The thoracodorsal artery perforator (TDAP) flap, first described as a latissimus dorsi musculocutaneous flap without muscle [4], established that a skin paddle could be harvested on thoracodorsal perforators without sacrificing the underlying LD muscle, and cadaveric study has shown the thoracodorsal artery to bifurcate consistently into horizontal and descending branches on the deep surface of the muscle, with the majority of musculocutaneous perforators arising from the descending branch [5]. Subsequently, muscle-sparing LD flap techniques were developed to preserve most of the LD muscle and its innervation while retaining a limited muscle segment around the vascular pedicle [6,7].

Several configurations of muscle-sparing LD flaps have been described. Schwabegger et al. demonstrated that preservation of the majority of the LD muscle and its motor innervation could reduce functional and aesthetic donor-site morbidity [6]. Saint-Cyr et al. subsequently described a descending-branch muscle-sparing LD flap based on the intramuscular branching pattern of the thoracodorsal system, reporting reliable flap survival with minimal functional impairment [7]. Extended transverse skin paddles have also been investigated in muscle-sparing LD flaps, demonstrating the ability of the thoracodorsal vascular system to support relatively extensive skin territories [8]. In these descriptions, however, the retained muscle is conceived primarily as a cuff surrounding the vascular pedicle, and the reported skin paddles have been oriented transversely. Neither the descending-branch approach nor the preservation of residual innervation is claimed as novel here.

Colohan et al. characterized the vascular anatomy of the descending-branch muscle-sparing flap in cadaveric and angiographic studies and applied it clinically as a free flap in five patients [9]. Vertically oriented muscle-sparing LD flaps have also been described for specific indications, including implant-based breast reconstruction without intraoperative position change [10] and reconstruction of radiation-induced chest wall ulcers [11]. These reports establish the feasibility of a vertical skin-paddle orientation but address defects of the breast and chest wall, and none defines the extent of the retained muscle in relation to reproducible intraoperative landmarks.

The configuration described in the present study instead treats the retained muscle as a longitudinal carrier and follows three design principles: the muscle segment is made long enough to support perfusion along an elongated paddle, it is oriented parallel to the long axis of the skin paddle rather than confined to the pedicle entry point, and its width is kept to the minimum required to contain the descending branch. The intent is to trade muscle length for muscle volume—accepting a longer strip of muscle in order to keep it narrow—so that longitudinal vascular continuity is obtained without the muscle bulk or the extensive intramuscular perforator dissection that the alternatives require. The clinical behavior of this configuration beneath a long, relatively narrow skin paddle has not been characterized in a consecutive series.

In our practice, we have used a modified muscle-sparing LD flap in which a narrow longitudinal cuff of LD muscle is incorporated along the long axis of the skin paddle. Rather than serving primarily as a source of tissue volume, the retained muscle cuff is intended to act as a vascular carrier along the longitudinal axis of the flap while preserving most of the LD muscle and, whenever feasible, its motor nerve branches. This configuration was developed as a practical strategy for harvesting elongated skin paddles without extensive intramuscular perforator dissection. The purpose of this study was to describe this technique and evaluate its clinical feasibility and reliability, with particular emphasis on flap dimensions, operative time, flap survival, distal skin-paddle complications, and donor-site outcomes.

## 2. Materials and Methods

### 2.1. Study Design and Patients

This retrospective case-series study included consecutive patients who underwent soft-tissue reconstruction using an LD flap with a longitudinally oriented muscle cuff at Hanyang University Hospital between March 2020 and August 2025. The study protocol was approved by the Institutional Review Board of Hanyang University Hospital (IRB No. 2026-07-007, approved 20 July 2026), which waived the requirement for informed consent given the retrospective design. All operations were performed as part of routine clinical care, independently of and prior to the conception of this study; the institutional review board approval was obtained subsequently for the retrospective review of existing medical records, and no data were extracted before approval was granted.

Patients were eligible for inclusion if reconstruction was performed using an LD flap incorporating a longitudinal muscle component beneath an elongated skin paddle. Flaps were included regardless of the width of the muscle cuff, spanning muscle-sparing LD (msLD) harvests and, in cases in which the cuff extended proximal to the thoracodorsal bifurcation, musculocutaneous LD (mcLD) harvests. 

Twenty-eight consecutive patients who underwent reconstruction with this technique during the study period were initially identified. Three were excluded: two for insufficient operative records and one for postoperative follow-up shorter than 3 months. The remaining 25 patients constituted the study cohort and represent all eligible consecutive cases performed during the study period. None of these 25 patients has been reported in any previous publication. One of the authors has co-authored a previous case report of a vertically oriented muscle-sparing LD flap [11]; that report described two patients treated at a different institution for radiation-induced chest wall ulcers following breast cancer surgery, and there is no overlap with the present cohort. The other previously reported series of vertical or descending-branch muscle-sparing LD flaps cited here [9,10] were performed at institutions with which the authors have no affiliation.

The decision to use this flap rather than an alternative was made qualitatively at the time of surgery. This configuration was favored when the defect was substantially longer than it was wide, such that the required coverage could be obtained by adding length rather than width; when a thin, pliable paddle with a long reach was preferred over the bulk of a conventional musculocutaneous LD flap; and when the anticipated paddle length exceeded what was judged reliably supportable by a single thoracodorsal perforator, making a purely perforator-based TDAP harvest less attractive. An ALT flap was generally preferred when the recipient site was in the ipsilateral lower extremity and a supine single-position or two-team harvest was advantageous, or when thigh donor-site morbidity was considered preferable to a back donor site. These indications reflect institutional practice rather than a formal algorithm.

For the purposes of this study, flaps were classified as msLD or mcLD flaps according to the extent of muscle harvest and preservation of the thoracodorsal motor nerve branches. In the msLD flap, only a limited longitudinal segment of the LD muscle was harvested; the horizontal branch of the thoracodorsal nerve was preserved, whereas the descending branch was sacrificed. In contrast, the mcLD flap involved a relatively greater amount of muscle harvest, with sacrifice of both the horizontal and descending branches of the thoracodorsal nerve.

### 2.2. Surgical Technique

The flap was harvested with the patient in supine position, preferentially from the right lateral thoracic region. The ipsilateral arm was abducted to approximately 90°, and a small cushion was placed beneath the scapula to slightly elevate the operative side and facilitate exposure of the lateral border of the LD muscle.

The lateral border of the LD muscle was identified by palpation and pinching, and a skin incision was made approximately 2 cm anterior and parallel to the lateral muscle border. The incision was carried through the subcutaneous tissue, and the lateral border of the LD muscle was identified directly. Dissection was then continued medially in the plane between the LD and serratus anterior muscles. The descending branch of the thoracodorsal artery and its accompanying veins, together with the corresponding thoracodorsal motor nerve branch, were identified. The vascular pedicle and motor nerve were carefully separated, and the thoracodorsal vessels were dissected proximally until an adequate pedicle length was obtained.

The cuff was then designed between two landmarks on the descending branch. Distally, the reference was the level at which the branch could no longer be traced by direct vision on the deep surface of the muscle, either entering the muscle substance or becoming too fine to follow; proximally, the reference was the bifurcation. From the distal landmark, 10 cm was measured proximally along the vessel and the cuff was centered on this axis. This length was fixed by the vascular anatomy rather than scaled to the paddle, and the same approximately 10 cm cuff was used for paddles of 24 to 35 cm, on the rationale that the cuff acts as a conduit carrying the axial vessel and its perforators into the paddle rather than as a perfusion source proportional to its own volume.

For the msLD flap, a narrow longitudinal segment of the LD muscle, approximately 10 × 3 cm, was incorporated around the descending branch of the thoracodorsal vessels. The descending motor nerve branch supplying the harvested muscle segment was sacrificed, whereas the horizontal motor nerve branch was preserved to maintain innervation of the residual LD muscle. Thus, only a limited portion of muscle surrounding the longitudinal vascular axis was included in the flap.

For the mcLD flap, a relatively larger segment of the LD muscle, approximately 10 × 5 cm, was harvested. The muscle component included the branching point of the descending and horizontal branches of the thoracodorsal vascular system. In these cases, both the descending and horizontal motor nerve branches were sacrificed.

The distinction between the two configurations was therefore anatomically determined rather than electively chosen. Once the 10 cm cuff had been marked proximally from the distal landmark, the position of its proximal end relative to the bifurcation dictated the configuration. If the proximal end of the cuff fell distal to the bifurcation, the horizontal branch and its motor nerve lay outside the cuff and were preserved, and the flap was harvested as an msLD flap with a cuff of approximately 10 × 3 cm. If the proximal end of the cuff extended proximal to the bifurcation, the horizontal branch was necessarily contained within the cuff and its motor nerve was divided, and the flap was harvested as an mcLD flap with a cuff of approximately 10 × 5 cm. Which configuration resulted in a given patient thus depended on the distance between the distal landmark and the bifurcation, which varies between individuals; it was not determined by the dimensions of the skin paddle or by the requirements of the recipient site. This accounts for the predominance of msLD harvests (21 of 25, 84.0%) and for the four mcLD harvests (16.0%) in this series.

**Figure 1:**
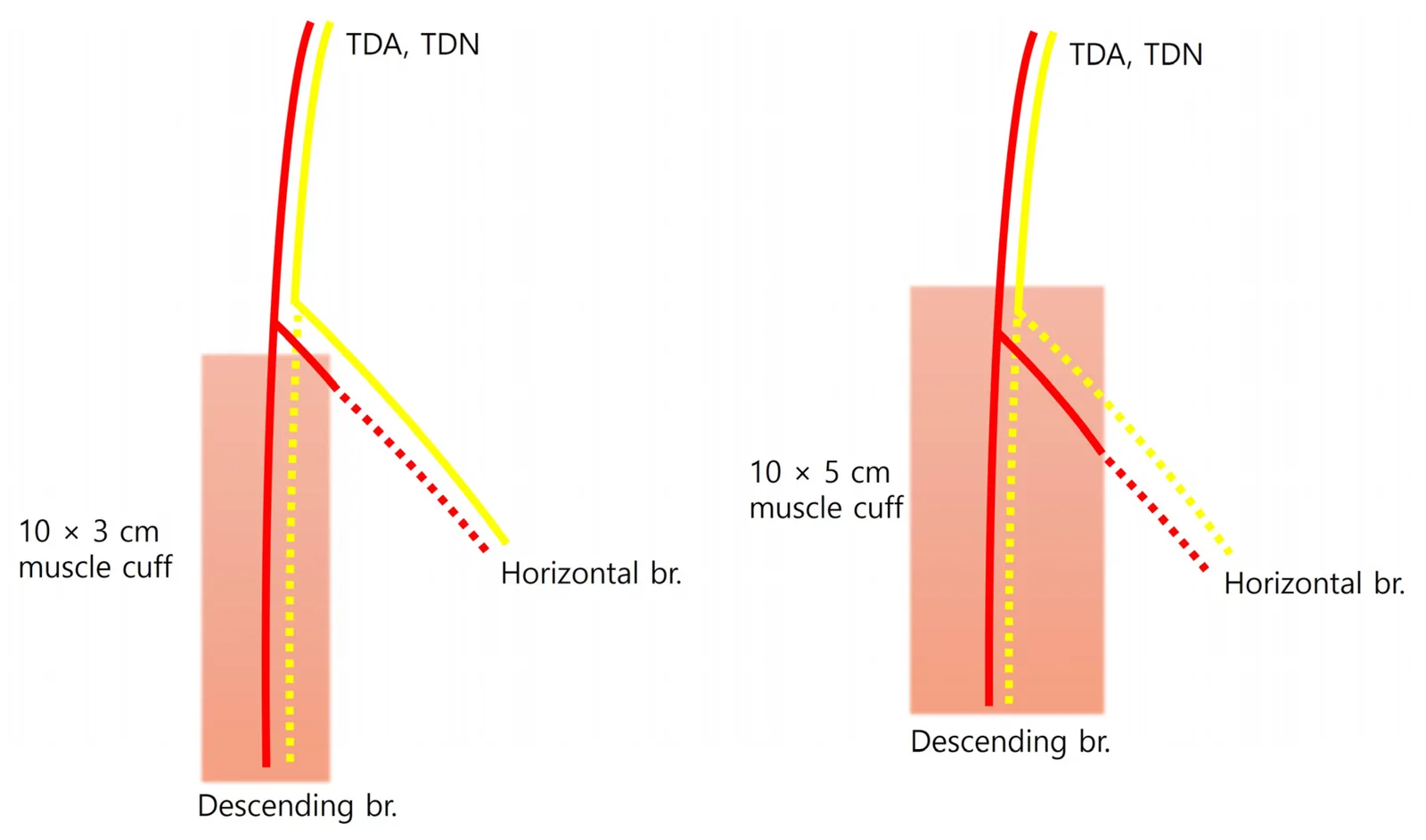
Schematic representation of the longitudinal muscle-cuff configuration and of the anatomical basis for the msLD and mcLD variants. The thoracodorsal artery (TDA) and thoracodorsal nerve (TDN) run together and divide into descending and horizontal branches. Red indicates the vascular system: solid red denotes vessels incorporated into the flap, and dotted red denotes vessels that were ligated. Yellow indicates the motor nerve: solid yellow denotes nerve branches left functionally intact, and dotted yellow denotes branches that were divided. The shaded rectangle denotes the muscle cuff harvested with the flap. The distal margin of the cuff corresponds to the level at which the descending branch can no longer be followed by direct vision on the undersurface of the latissimus dorsi, either because it enters the muscle substance or because it becomes too fine to identify; from this level, a cuff carrying 10 cm of the descending branch is marked in the proximal direction. Left: when the proximal margin of the cuff lies distal to the bifurcation of the thoracodorsal vessels, the horizontal branch lies outside the cuff and its motor nerve is preserved, yielding a muscle-sparing LD (msLD) flap with an approximately 10 × 3 cm cuff. Right: when the proximal margin extends proximal to the bifurcation, the horizontal branch is necessarily included and its motor nerve is divided, yielding a musculocutaneous LD (mcLD) flap with an approximately 10 × 5 cm cuff. The resulting configuration is therefore determined by the distance between the distal margin and the bifurcation in the individual patient rather than by the dimensions of the skin paddle.

In both flap types, the muscle component was oriented longitudinally beneath the skin paddle (Figure 1). The skin paddle was designed according to the dimensions of the recipient-site defect, with emphasis on obtaining sufficient longitudinal reach while limiting unnecessary flap width. The cutaneous perforators arising from the descending branch were not skeletonized individually; instead, they were left undisturbed within the retained muscle strip and elevated en bloc with it, so that vascular continuity between the axial vessel and the skin paddle was maintained without intramuscular dissection. This is the principal technical difference from a perforator-based harvest, in which each perforator is traced through the muscle and the muscle itself is left in situ.

After flap elevation, the flap was transferred to the recipient site, and microvascular anastomosis was performed using suitable recipient vessels. The donor site was closed primarily whenever closure could be achieved without excessive tension. When primary closure was not feasible, a split-thickness skin graft was used.

### 2.3. Data Collection and Outcome Measures

Demographic and clinical data were retrospectively extracted from medical records and operative reports. The collected variables included age, sex, body mass index (BMI), comorbidities, etiology and location of the defect, flap type, and additional reconstructive procedures. A patient-level summary of all collected variables is provided in Table S1 (Supplementary Materials).

Flap-related variables included skin-paddle length and width, calculated flap surface area, length-to-width ratio, operative time, and donor-site closure method. Flap area was calculated as the product of the maximum recorded long and short axes of the skin paddle. The length-to-width ratio was calculated by dividing the long axis by the short axis.

The primary outcome was flap survival, including total flap loss and partial skin-paddle necrosis. Partial necrosis was defined as full-thickness necrosis of a portion of the skin paddle, irrespective of whether operative debridement was required. Total flap loss was defined as necrosis of the entire flap. When partial necrosis occurred, its location, dimensions, management, and final outcome were recorded from operative notes, outpatient records, and postoperative photographs. Secondary outcomes included donor-site closure method, donor-site complications, requirement for additional surgical intervention, operative time, and duration of postoperative follow-up. Donor-site functional morbidity was retrospectively assessed from outpatient follow-up records, including documented complaints of shoulder pain, weakness, limitation of motion, or difficulty with overhead activities. When available, clinical examination findings and postoperative photographs were also reviewed for active elevation of the ipsilateral upper extremity.

Upper-extremity function was assessed using the Quick Disabilities of the Arm, Shoulder and Hand (QuickDASH) questionnaire, a validated 11-item patient-reported outcome measure scored from 0 (no disability) to 100 (maximum disability). The questionnaire is administered routinely at the 3-month postoperative outpatient visit at our institution, and completed forms were available for all 25 patients; the scores were retrieved retrospectively from these records together with the other study variables. Because the questionnaire is not administered preoperatively, the scores represent a cross-sectional assessment of postoperative upper-extremity function at 3 months rather than a measure of change from baseline.

Operative time was defined as the interval from the initial skin incision for recipient-vessel preparation to completion of the final wound closure, and therefore encompassed the entire reconstructive procedure: recipient-vessel preparation, flap harvest and donor-site closure, and flap insetting with microvascular anastomosis. It did not include anesthetic induction, positioning, or preparation before incision.

### 2.4. Subgroup Analysis According to Skin-Paddle Geometry

For exploratory analysis, a length-to-width ratio of 2.5 was used to divide the cohort into relatively broad and elongated flap configurations. Patients were accordingly categorized into two groups: a length-to-width ratio <2.5 and ≥2.5.

Patient age, BMI, flap dimensions, flap area, operative time, flap type, donor-site closure method, flap-related complications, and follow-up duration were compared between the two groups. This subgroup analysis was considered exploratory and hypothesis-generating only. The cutoff of 2.5 was not derived from prior literature or from a predefined hypothesis but was selected post hoc as a descriptive division of the observed distribution. Because the ratio is itself a function of paddle width and length, comparisons of width and area between the groups are structurally dependent on the grouping variable and are reported as descriptive rather than independent findings. Given the limited sample size, the number of comparisons performed, and the absence of adjustment for multiple testing, all *p*-values are regarded as unadjusted and exploratory.

### 2.5. Statistical Analysis

Continuous variables are presented as mean ± standard deviation and range, and categorical variables are presented as numbers and percentages. Descriptive statistics were used to summarize demographic characteristics, flap dimensions, operative variables, flap-related outcomes, and follow-up duration.

For the exploratory comparison between the length-to-width ratio groups (<2.5 vs. ≥2.5), continuous variables were compared using Welch’s t-test, and categorical variables were compared using Fisher’s exact test. No adjustment for multiple comparisons was performed because of the exploratory nature and limited sample size of the study. A two-sided *p*-value < 0.05 was considered statistically significant.

Statistical analyses were performed using IBM SPSS Statistics for Windows, version 29.0 (IBM Corp., Armonk, NY, USA).

## 3. Results

### 3.1. Patient Characteristics

Of the 28 patients initially identified, 25 met the inclusion criteria and were analyzed (Table S1). The mean age was 54.0 ± 19.9 years (range, 20–89 years), and 13 patients (52.0%) were male. The mean body mass index was 25.0 ± 4.2 kg/m^2^ (range, 15.6–33.9 kg/m^2^). The most common etiology was scar contracture (14 patients, 56.0%), followed by osteomyelitis (5, 20.0%), trauma (4, 16.0%), burn injury (1, 4.0%), and tumor (1, 4.0%). The lower leg was the most common defect location (11 patients, 44.0%), followed by the thigh (5, 20.0%), foot and ankle (4, 16.0%), chest (2, 8.0%), upper extremity (2, 8.0%), and neck (1, 4.0%) (Table 1).

### 3.2. Flap Characteristics and Clinical Outcomes

Twenty-one flaps (84.0%) were classified as msLD flaps, and four (16.0%) were mcLD flaps. The mean long axis of the skin paddle was 26.8 ± 3.2 cm (range, 24–35 cm), and the mean short axis was 11.4 ± 1.8 cm (range, 9–16 cm). The mean calculated skin-paddle area was 305.3 ± 63.8 cm^2^ (range, 216–528 cm^2^). The mean operative time was 154.8 ± 49.7 minutes (range, 70–300 minutes).

Primary donor-site closure was achieved in 14 patients (56.0%), whereas 11 patients (44.0%) required split-thickness skin grafting. Partial flap necrosis occurred in two patients (8.0%), and no total flap loss occurred. In the first patient, who underwent chest reconstruction, necrosis measuring 5 × 1 cm developed at the distal margin of the skin paddle and was managed by excision of the necrotic segment and re-suturing, with subsequent uneventful healing. In the second patient, who underwent foot reconstruction, necrosis measuring 2 × 1 cm developed at the distal margin and was managed conservatively by secondary healing without operative intervention, also with complete healing. In both cases the necrosis was confined to the distal portion of the elongated paddle, the remainder of the flap survived, and no additional flap surgery was required. The mean follow-up duration was 15.0 ± 8.8 months (range, 3–36 months) (Table 2).

Donor-site complications were reviewed individually. One patient (4.0%) developed partial wound dehiscence at a primarily closed donor site, which was managed by re-suturing and healed without further sequelae. No other donor-site complication was documented: there were no cases of seroma, hematoma, or surgical-site infection; no partial or complete loss of the split-thickness skin grafts used for donor-site coverage and no graft-related complications among the 11 grafted donor sites; no delayed wound healing; and no hypertrophic scarring recorded during follow-up. Apart from the single re-suturing described above, no patient required a secondary procedure at the donor site.

QuickDASH scores were obtained from all 25 patients. The mean score was 2.82 ± 3.10 (median, 2.27; interquartile range, 0–4.55; range, 0–11.36) on a 0–100 scale. Nine patients (36.0%) reported a score of 0, indicating no perceived upper-extremity disability, and the highest score recorded was 11.36. No patient reported a score above 12. With respect to donor-site function, no patient reported shoulder pain or weakness, and no restriction of shoulder range of motion was documented at any follow-up visit.

### 3.3. Comparison of Skin-Paddle Length-to-Width Ratio

For exploratory analysis, patients were divided into two groups according to a skin-paddle length-to-width ratio of 2.5: 12 patients had a ratio <2.5, and 13 had a ratio ≥2.5. The mean length-to-width ratio was 2.0 ± 0.2 in the <2.5 group and 2.7 ± 0.4 in the ≥2.5 group.

The mean long-axis length did not differ between the two groups (26.2 ± 2.3 cm vs. 27.3 ± 3.9 cm, *p* = 0.38). Because long-axis length was comparable, the ≥2.5 group necessarily comprised narrower paddles (10.0 ± 0.4 cm vs. 12.9 ± 1.5 cm) with a correspondingly smaller calculated area (273.5 ± 43.7 cm^2^ vs. 339.7 ± 65.6 cm^2^); these differences follow arithmetically from the definition of the groups and are therefore reported descriptively rather than as independent findings.

There were no differences in age, BMI, operative time, or flap type between the two groups. Primary donor-site closure was achieved more frequently in the ≥2.5 group than in the <2.5 group (76.9% vs. 33.3%, *p* = 0.047, unadjusted). Because the ≥2.5 group consisted of narrower paddles by definition, and paddle width is the principal determinant of whether the donor site can be closed directly, this association cannot be separated from the variable used to define the groups. Partial flap necrosis occurred in one patient in each group (8.3% vs. 7.7%, *p* = 1.00), and no total flap loss occurred in either group. Follow-up duration was also comparable between groups (12.5 ± 6.0 months vs. 17.3 ± 10.5 months, *p* = 0.17) (Table 3).

### 3.4. Case Presentations

#### 3.4.1. Case 1: Musculocutaneous LD (mcLD) Flap

A 56-year-old woman with extensive post-burn scar contracture of the chest underwent reconstruction using an mcLD flap. A 27 × 12 cm skin paddle was harvested together with an approximately 10 × 5 cm longitudinal muscle component. Because the defect exceeded the dimensions of the flap after complete scar release, split-thickness skin grafting was additionally performed at three residual areas.

**Figure 2:**
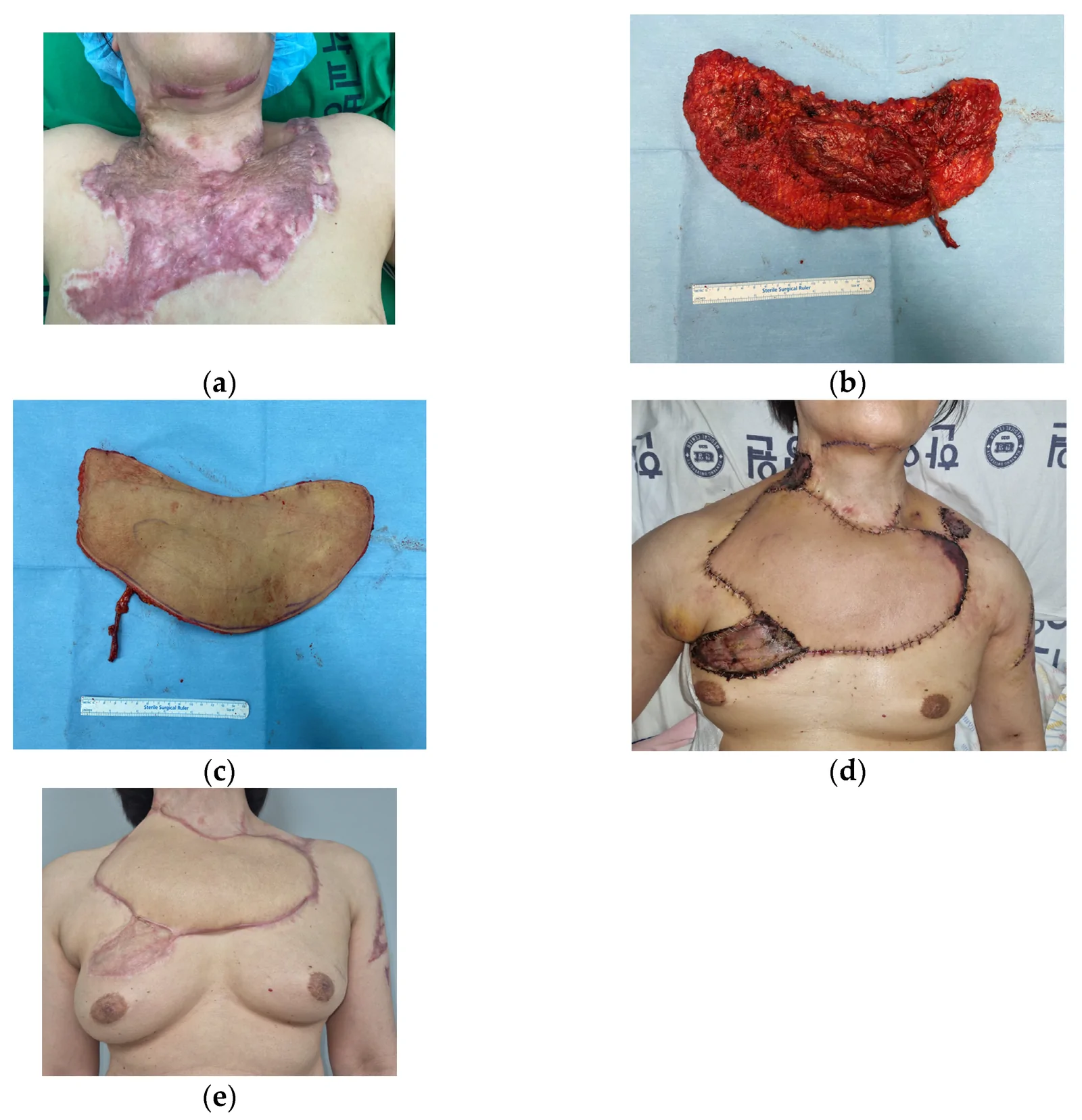
Musculocutaneous LD (mcLD) flap reconstruction of extensive post-burn scar contracture of the anterior chest and neck in a 56-year-old woman. (**a**) Intraoperative view before scar release, showing extensive hypertrophic scar contracture involving the anterior neck and upper chest. (**b**) Harvested flap, muscle side: a 27 × 12 cm skin paddle elevated with an approximately 10 × 5 cm longitudinal muscle component oriented along the long axis of the paddle. (**c**) The same flap viewed from the skin side. (**d**) Postoperative day 5, showing necrosis measuring 5 × 1 cm limited to the left distal tip of the skin paddle; residual defects not covered by the flap after complete scar release were managed with split-thickness skin grafting. (**e**) One year after excision of the necrotic tip and re-suturing, showing stable soft-tissue coverage; the patient reported no functional discomfort related to contracture.

An area of distal flap-tip necrosis measuring 5 × 1 cm developed at the distal margin (left distal tip) of the skin paddle. The necrotic tissue was excised, and the wound was successfully managed by re-suturing without further flap-related complications. The operative time was 140 minutes, and a stable reconstructive result was maintained at 12 months of follow-up (Figure 2).

#### 3.4.2. Case 2: Muscle-Sparing LD (msLD) Flap

A 74-year-old woman with hypertension, dyslipidemia, rheumatoid arthritis, and chronic osteomyelitis of the lower leg underwent reconstruction using a muscle-sparing latissimus dorsi flap. A long and narrow skin paddle measuring 30 × 10 cm was harvested together with an approximately 10 × 3 cm longitudinal muscle cuff based on the descending branch of the thoracodorsal vascular system. The horizontal motor nerve branch was preserved in accordance with the muscle-sparing configuration.

**Figure 3:**
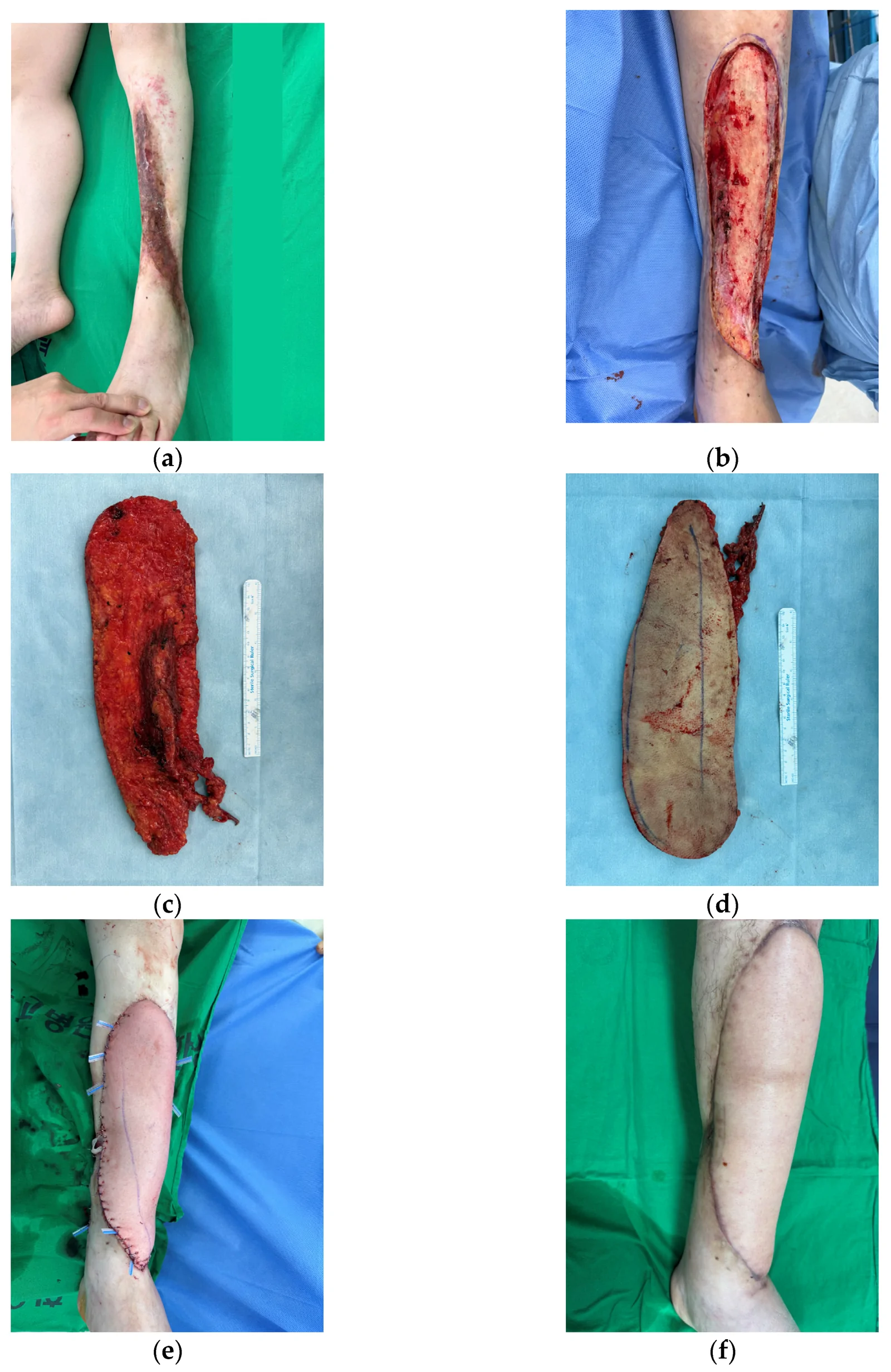
Muscle-sparing LD (msLD) flap reconstruction of chronic osteomyelitis of the lower leg in a 74-year-old woman. (**a**) Preoperative view of the lower leg, showing chronic osteomyelitis with overlying unstable scarred soft tissue. (**b**) After radical debridement, leaving an elongated soft-tissue defect along the long axis of the leg. (**c**) Harvested flap, muscle side: a 30 × 10 cm skin paddle elevated with an approximately 10 × 3 cm longitudinal muscle cuff based on the descending branch of the thoracodorsal vessels, oriented along the long axis of the paddle. (**d**) The same flap viewed from the skin side, demonstrating the long, relatively narrow paddle design. (**e**) Immediate postoperative view after inset and microvascular anastomosis, showing a well-perfused flap; the donor site was closed primarily. (**f**) One year postoperatively, showing stable soft-tissue coverage with satisfactory contour and no evidence of recurrent infection.

The donor site was closed primarily. The operative time was 70 minutes, and the flap survived completely without flap-related complications. During 24 months of follow-up, the reconstructed lower leg demonstrated stable soft-tissue coverage and satisfactory contour (Figure 3).

#### 3.4.3. Case 3: Musculocutaneous LD (mcLD) Flap for a Chronic Unstable Wound of the Lower Leg

**Figure 4:**
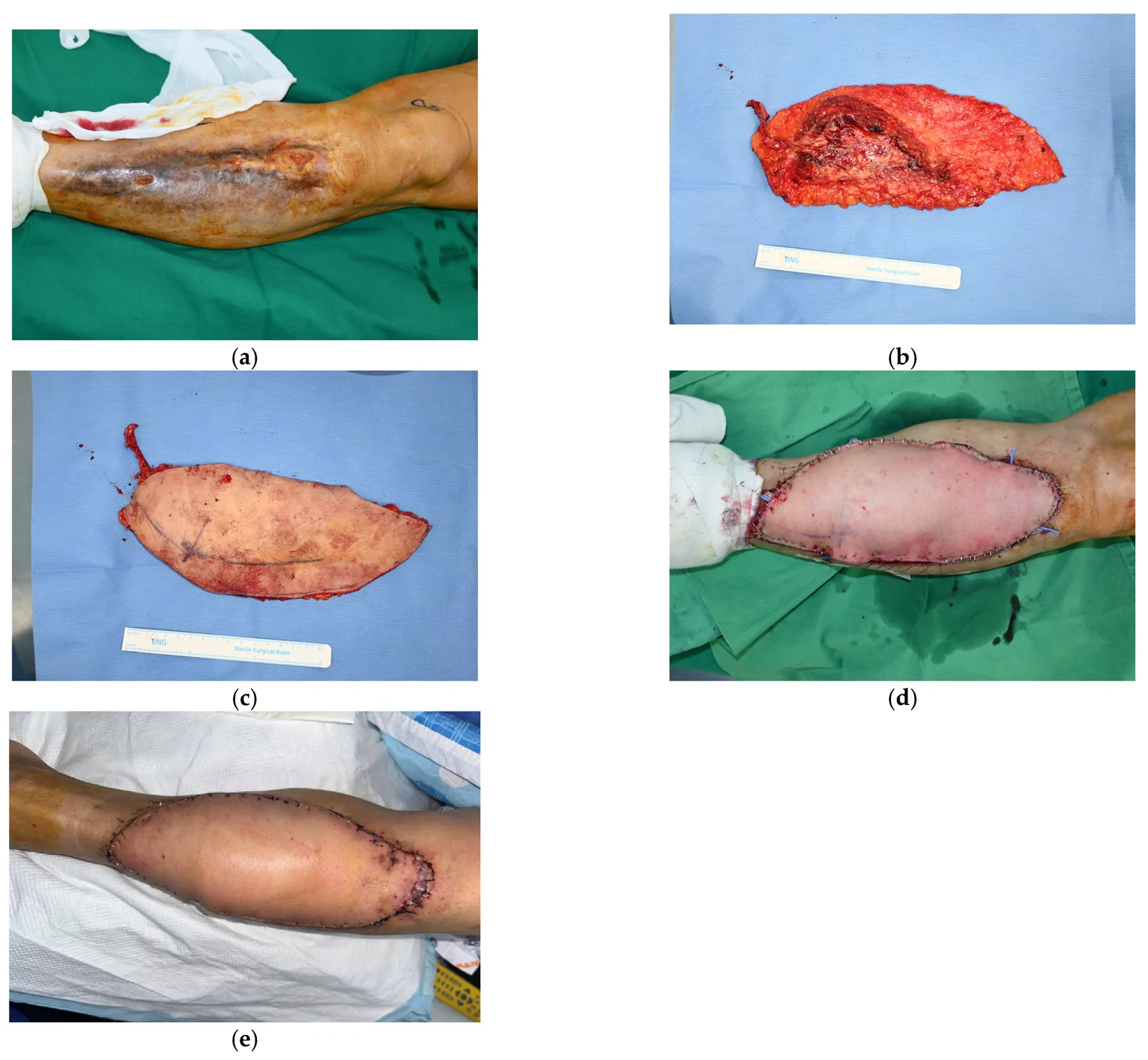
Musculocutaneous LD (mcLD) flap reconstruction of a chronic unstable wound of the lower leg in a 75-year-old man. (**a**) Preoperative view showing a chronic unstable wound extending along the long axis of the lower leg, with unstable scarring and focal ulceration. (**b**) Harvested flap, muscle side: an approximately 10 × 5 cm longitudinal muscle cuff oriented along the long axis of the skin paddle, encompassing the bifurcation of the thoracodorsal vessels. (**c**) Harvested flap viewed from the skin side, demonstrating the long, relatively narrow 25 × 10 cm paddle design. (**d**) Immediate postoperative view after inset and microvascular anastomosis, showing a well-perfused 25 × 10 cm skin paddle covering the elongated defect. (**e**) One month postoperatively, showing a healed wound with stable soft-tissue coverage.

A 75-year-old man with a chronic unstable wound of the lower leg underwent reconstruction with a musculocutaneous latissimus dorsi flap. A 25 × 10 cm skin paddle was harvested together with an approximately 10 × 5 cm longitudinal muscle cuff; because the cuff extended proximal to the thoracodorsal bifurcation, the horizontal branch was included and its motor nerve was divided. The donor site required split-thickness skin grafting. The operative time was 240 minutes, and the flap survived completely without flap-related complications. The wound had healed by 1 month postoperatively, and stable soft-tissue coverage of the elongated defect was maintained over 12 months of follow-up (Figure 4).

## 4. Discussion

The present study describes a longitudinal muscle-cuff configuration of the LD flap for reconstruction requiring long and relatively narrow skin paddles. In this series of 25 patients, the mean skin-paddle dimensions were 26.8 × 11.4 cm, corresponding to a mean calculated area of 305.3 cm^2^, and 21 flaps (84.0%) were harvested in a muscle-sparing configuration, with the wider mcLD cuff resulting in the four cases in which the 10-cm cuff extended proximal to the thoracodorsal bifurcation. No total flap loss occurred, and partial necrosis was limited to two patients (8.0%), both of small extent. These results indicate that harvest of elongated LD skin paddles over a longitudinally oriented muscle component is feasible and was associated with favorable flap survival in this cohort, while limiting the amount of muscle incorporated into the flap. Because no comparator group with a similar paddle geometry harvested without a muscle cuff was available, these observations describe clinical outcomes rather than a demonstrated perfusion mechanism.

Large perforator-based flaps have become indispensable for coverage of extensive soft-tissue defects, and the ALT flap offers a wide skin territory and considerable freedom in flap design. However, perfusion reliability becomes an important consideration as the skin paddle extends farther from the dominant perforator. The perforasome concept has demonstrated that individual perforators supply distinct vascular territories connected through direct and indirect linking vessels, with vascular density generally decreasing toward the periphery of the perforasome [12]. In a clinical series of 303 ALT free flaps for lower-extremity reconstruction, Min et al. reported a 14.2% rate of partial flap loss and identified greater distance from the perforator to the flap margin and larger flap size as independent risk factors for partial necrosis [13]. Although large skin paddles may survive on a single dominant perforator through recruitment of adjacent perforasomes [12], extended designs may benefit from the inclusion of additional perforators or vascular augmentation in selected cases to enhance perfusion reliability. These strategies, however, may increase the complexity of intramuscular dissection and microvascular reconstruction. Similar considerations apply to abdominal perforator flaps. When perfusion from a single pedicle is considered insufficient, a bipedicled DIEP flap may be used to augment vascular supply, although this requires additional pedicle dissection and has been associated with longer operative times [14].

The thoracodorsal vascular system offers a different solution to the same problem. Conventional musculocutaneous LD flaps are well recognized for their dependable vascularity and capacity to provide large volumes of vascularized tissue, but this reliability is obtained at the cost of substantial muscle sacrifice and potential donor-site functional morbidity [3]. The TDAP flap showed that the skin territory of the LD region could be transferred with complete muscle preservation [5], and muscle-sparing LD techniques subsequently established an intermediate strategy in which only a limited muscle segment around the thoracodorsal branches is harvested, maintaining reliable perfusion while preserving most of the muscle and its motor innervation [6,7].

The distinguishing feature of this technique is the geometry of the retained muscle rather than the vascular branch on which it is based. Conventional muscle-sparing designs retain a compact cuff around the pedicle and derive perfusion of the paddle from that single point of entry; here the cuff is extended along the descending branch and aligned with the long axis of the paddle, so that vascular continuity is distributed along the length of the flap. Because the cuff is narrow—approximately 3 cm in the msLD configuration—the retained volume remains modest despite the greater length: length is substituted for bulk rather than added to it. The corollary is that the extent of harvest is set by the vascular anatomy rather than by the reconstructive requirement, and msLD and mcLD are the two possible outcomes of applying the same rule, which accounts for the 21:4 distribution in this series. In the muscle-sparing configuration, an approximately 10 × 3 cm muscle segment is retained beneath the long axis of the skin paddle while preserving the horizontal motor nerve branch. The consistent perforator locations described along the descending thoracodorsal branch provide a plausible anatomical rationale for this design [15], although the present study did not assess perfusion of vertically oriented skin paddles directly. Earlier reports of muscle-sparing LD flaps have focused on extended transverse skin paddles [8], whereas the present configuration applies the same thoracodorsal vascular axis in the longitudinal direction to obtain length rather than width.

Within the limits of a post hoc division of a 25-patient cohort, greater skin-paddle elongation was not accompanied by an apparent increase in flap-related complications: partial necrosis occurred in one patient in each group (8.3% in the <2.5 group vs. 7.7% in the ≥2.5 group), and no total flap loss occurred in either group. Because the mean long-axis length was similar between groups (26.2 vs. 27.3 cm), the ≥2.5 group by construction comprised narrower paddles (12.9 vs. 10.0 cm) of smaller calculated area (339.7 vs. 273.5 cm^2^); these contrasts describe the grouping variable rather than an independent effect of elongation. The clinically useful observation is therefore descriptive: comparable longitudinal reach was achieved with a narrower paddle, and this was not accompanied by a higher observed rate of distal necrosis in this small series.

This geometric characteristic also has implications for the donor site. Primary closure was achieved in 76.9% of patients with a length-to-width ratio ≥2.5 compared with 33.3% of those with a lower ratio (*p* = 0.047, unadjusted). This difference should not be read as an independent benefit of an elongated design: the ≥2.5 group consisted of narrower paddles by definition, and donor-site width is the principal determinant of direct closure, so the association is confounded by the grouping variable itself. What the data support is the more limited and largely mechanical point that a narrower donor defect is easier to close directly—a consideration relevant in extremity reconstruction, where defects are frequently elongated rather than broadly transverse, and where the required coverage can often be obtained by adding length rather than width.

This point should not obscure the fact that 11 of 25 donor sites (44.0%) could not be closed primarily and required split-thickness skin grafting. Donor-site morbidity of this magnitude is consistent with reports of large thoracodorsal-based harvests for extensive extremity defects, in which major donor-site complications occurred in 13% of 69 patients [16]. A grafted back donor site carries its own morbidity—a second wound at the graft harvest site, a longer period of dressing care, and a residual contour and pigmentation difference that is cosmetically inferior to a linear scar—and this proportion is therefore a substantive limitation of the approach rather than an incidental detail. It also qualifies the muscle-sparing rationale: limiting the volume of muscle harvested reduces the functional cost of the donor site but does not reduce the cutaneous deficit, which is set by the width of the skin paddle. In practical terms it is paddle width, not the extent of muscle harvest, that governs whether primary closure is achievable. Although all grafts healed without complication in this cohort, a design requiring grafting in nearly half of cases leaves clear room for improvement, and preoperative counselling should reflect this likelihood. Seroma is the principal determinant of graft healing at this donor site, with rates as high as 69.2% reported after extended latissimus dorsi harvest [17], and negative-pressure dressing has been shown to improve graft take and reduce seroma compared with conventional bolster dressing [18]; both represent avenues for reducing the burden of the grafted donor site in this configuration.

The muscle-sparing configuration resulted in most patients in this series, whereas the mcLD configuration arose in the minority in whom the 10-cm cuff extended proximal to the thoracodorsal bifurcation. In the msLD flap, preservation of the horizontal motor nerve branch maintains innervation of the residual muscle, while only the descending branch supplying the harvested cuff is sacrificed. The musculocutaneous configuration incorporates a larger muscle segment around the branching point of the descending and horizontal vascular branches and therefore requires sacrifice of both corresponding motor nerve branches. The functional consequence of this distinction is therefore set by the individual vascular anatomy; in either configuration, the extent of muscle harvest remains substantially less than that of a conventional LD flap, in which the entire muscle is sacrificed.

Consistent with this muscle-sparing rationale, clinically significant donor-site functional complaints were not documented during routine follow-up. Patients generally retained the ability to actively elevate the ipsilateral upper extremity on clinical observation. These observations do not substitute for formal functional testing and should not be equated with demonstrated preservation of shoulder function; anatomical preservation of the horizontal motor branch was confirmed intraoperatively, whereas its functional consequence was not measured. Accordingly, these findings indicate only that the muscle-sparing harvest was technically feasible and that no functional complaints were recorded during routine follow-up.

To address this more directly, QuickDASH was obtained from the entire cohort at 3 months postoperatively. The mean score was 2.82 and the median 2.27 (interquartile range, 0–4.55), with 36.0% of patients scoring 0. For comparison, normative data from 961 Japanese volunteers without upper-limb treatment reported a median QuickDASH of 2.3 (interquartile range, 0–6.8) and a mean of 4.8 ± 7.8 [19]. The present cohort therefore scored at essentially the same median and a lower mean than an untreated reference population of comparable age, indicating that clinically meaningful patient-perceived upper-extremity disability was uncommon after this harvest—a finding consistent with anatomical preservation of the horizontal motor branch in the msLD configuration. Two qualifications remain. First, the questionnaire was administered postoperatively only, so the scores describe the functional state at 3 months rather than a change attributable to surgery; patients scoring low may have scored comparably low beforehand. Second, QuickDASH is a self-reported composite of upper-limb function and does not isolate latissimus dorsi-specific strength or shoulder range of motion, neither of which was measured objectively. Studies that have applied objective measures to this donor site—a randomized comparison of conventional and perforator-based thoracodorsal harvests using the Constant–Murley score [20], and a prospective dynamometric assessment of shoulder strength and range of motion after latissimus dorsi transfer [21]—indicate that patient-reported instruments alone may not capture the functional consequences of muscle harvest. The results are therefore best read as indicating that this harvest was not associated with substantial patient-perceived disability, rather than as a direct demonstration that shoulder function was preserved.

A further practical feature of this approach is the relative simplicity of the pedicle dissection. The descending thoracodorsal bundle is identified in the plane between the latissimus dorsi and serratus anterior muscles and dissected proximally without extensive intramuscular skeletonization of individual cutaneous perforators. The mean operative time was 154.8 minutes for the entire reconstructive procedure, from the initial incision for recipient-vessel preparation to final wound closure, and did not differ between the lower- and higher-ratio groups, suggesting that increasing elongation within the observed range imposed no obvious additional operational burden. Because no direct comparison with ALT, TDAP, or conventional LD flap harvest was performed, however, superiority in operative efficiency cannot be claimed.

Several limitations should be acknowledged. First, this was a retrospective case series with a small number of patients and no control group, and it was therefore designed to assess feasibility and clinical reliability rather than superiority over alternative options. Second, the length-to-width cutoff of 2.5 was chosen exploratively, and the subgroup analysis was not powered to detect differences in complication rates. Third, flap perfusion was assessed clinically, without intraoperative perfusion imaging or quantitative vascular measurement; consequently, although the favorable survival of elongated paddles supports the reliability of this configuration, a causal relationship between the longitudinal muscle cuff and improved distal perfusion cannot be established. Fourth, although QuickDASH scores were low across the cohort and no clinically significant donor-site functional complaints were identified during routine follow-up, the questionnaire was administered at a single postoperative time point without a preoperative baseline, and objective assessments of shoulder strength and range of motion were not performed. Preservation of shoulder function therefore cannot be definitively established from the present study, and the low scores should be read as indicating an absence of substantial patient-perceived disability rather than as evidence that function was unchanged by the operation. Fifth, the sample of 25 flaps is small and the cohort was heterogeneous in defect etiology and location, so the series cannot define the perfusion limits of this design or identify risk factors for distal necrosis; with only two events, the determinants of partial necrosis could not be analyzed. Prospective validation in a larger, preferably multicenter cohort, with objective perfusion assessment and standardized functional outcome measures, would be needed to confirm these observations. A larger series would also permit examination of the anatomical relationship between the site of partial necrosis and the distal segment of the elongated paddle, which may help establish the practical length limits of this design.

Despite these limitations, the present series demonstrates that a longitudinal muscle-cuff LD flap can provide long and relatively narrow skin paddles with a low rate of clinically relevant distal necrosis and no total flap loss. Preservation of the horizontal motor nerve branch in the muscle-sparing configuration allows most of the muscle and its innervation to be retained, while the longitudinal cuff offers a simple means of incorporating the thoracodorsal vascular axis beneath an elongated paddle. This technique may therefore represent a useful option when substantial longitudinal coverage is required and extensive perforator dissection or sacrifice of the entire latissimus dorsi muscle is undesirable.

## 5. Conclusions

Harvest of long and relatively narrow latissimus dorsi skin paddles over a longitudinal muscle cuff was feasible and was associated with favorable flap survival in this series, with no total flap loss and only limited partial necrosis. The muscle-sparing configuration allowed anatomical preservation of most of the LD muscle and its horizontal motor innervation while avoiding extensive intramuscular perforator dissection, although shoulder function was not objectively assessed. Within this cohort, the more elongated designs achieved comparable longitudinal coverage with a narrower paddle; the accompanying higher rate of primary donor-site closure is descriptive and is closely related to paddle width, which defined the groups. Taken together, these findings support the technical feasibility of this configuration for elongated soft-tissue defects, and comparative studies with objective perfusion and functional assessment are required to establish any advantage over alternative reconstructive options.

## Figures and Tables

**Table 1:** Patient demographics (*n* = 25).

Characteristic	Value (%)
No. of patients	25
Age, yr	54.0 ± 19.9
	(range, 20–89)
Sex	
Male	13 (52.0)
Female	12 (48.0)
Body mass index, kg/m^2^	25.0 ± 4.2
	(range, 15.6–33.9)
Comorbidities *	
Hypertension	8 (32.0)
Diabetes mellitus	4 (16.0)
Dyslipidemia	8 (32.0)
Others †	8 (32.0)
None	11 (44.0)
Etiologies	
Scar contracture	14 (56.0)
Osteomyelitis	5 (20.0)
Trauma	4 (16.0)
Burn	1 (4.0)
Tumor	1 (4.0)
Defect locations	
Lower leg	11 (44.0)
Thigh	5 (20.0)
Foot and ankle	4 (16.0)
Chest	2 (8.0)
Upper extremity	2 (8.0)
Neck	1 (4.0)

* Comorbidities are not mutually exclusive; a patient may have more than one. † Others included arteriovenous malformation, cerebrovascular accident, rheumatoid arthritis with interstitial lung disease, heart failure, hyperthyroidism, bronchiectasis, and malignancy.

**Table 2:** Perioperative flap characteristics and outcomes.

Characteristic	Value (%)
Flap type	
Muscle-sparing latissimus dorsi	21 (84.0)
Musculocutaneous latissimus dorsi	4 (16.0)
Skin flap long axis, cm	26.8 ± 3.2
	(range, 24–35)
Skin flap short axis, cm	11.4 ± 1.8
	(range, 9–16)
Skin flap area *, cm^2^	305.3 ± 63.8
	(range, 216–528)
Operation time, min	154.8 ± 49.7
	(range, 70–300)
Additional procedures at the recipient site	
None	14 (56.0)
Split-thickness skin graft	9 (36.0)
Deep inferior epigastric perforator flap	1 (4.0)
Chimeric flap	1 (4.0)
Donor-site closure	
Primary closure	14 (56.0)
Split-thickness skin graft	11 (44.0)
Flap-related complications	
Partial flap necrosis	2 (8.0)
Total flap loss	None
Donor-site complications	
Wound dehiscence	1 (4.0)
Seroma, hematoma, or infection	None
Graft loss or delayed healing	None
Secondary procedure required	1 (4.0)
Follow-up, mo	15.0 ± 8.8
	(range, 3–36)

* Skin flap area was calculated as the product of the long and short axes of the skin paddle.

**Table 3:** Comparison of outcomes by skin paddle length-to-width ratio.

Characteristic	Ratio < 2.5 (*n* = 12)	Ratio ≥ 2.5 (*n* = 13)	*p*-Value *
Length-to-width ratio	2.0 ± 0.2 (range, 1.7–2.5)	2.7 ± 0.4 (range, 2.5–3.5)	—
Skin flap long axis, cm	26.2 ± 2.3 (range, 25–33)	27.3 ± 3.9 (range, 24–35)	0.38
Skin flap short axis, cm	12.9 ± 1.5 (range, 11–16)	10.0 ± 0.4 (range, 9–11)	— †
Skin flap area, cm^2^	339.7 ± 65.6 (range, 275–528)	273.5 ± 43.7 (range, 216–350)	— †
Age, yr	46.8 ± 22.2	60.6 ± 15.5	0.09
Body mass index, kg/m^2^	25.3 ± 5.0	24.7 ± 3.4	0.76
Operation time, min	158.8 ± 55.7	151.2 ± 45.4	0.71
Musculocutaneous flap (mcLD)	2 (16.7)	2 (15.4)	1.00
Primary donor-site closure	4 (33.3)	10 (76.9)	0.047
Partial flap necrosis	1 (8.3)	1 (7.7)	1.00
Total flap loss	0 (0)	0 (0)	—
Follow-up, mo	12.5 ± 6.0	17.3 ± 10.5	0.17

Values are presented as mean ± standard deviation or number (%). * Continuous variables were compared using the Welch t-test and categorical variables using the Fisher exact test. † Skin flap short axis and calculated skin flap area are mathematically dependent on the length-to-width ratio used to define the groups; these variables are therefore presented descriptively and were not formally tested.

## Data Availability

The data presented in this study are available on request from the corresponding author. The data are not publicly available due to institutional privacy restrictions.

## Supplementary Materials

The following supporting information can be downloaded at: https://www.mdpi.com/article/10.3390/jcm15187296/s1. Table S1: Patient-level summary of latissimus dorsi flaps with a longitudinal muscle cuff (N = 25).

## Abbreviations

The following abbreviations are used in this manuscript:

ALT	Anterolateral thigh
BMI	Body mass index
DIEP	Deep inferior epigastric perforator
LD	Latissimus dorsi
mcLD	Musculocutaneous latissimus dorsi
msLD	Muscle-sparing latissimus dorsi
QuickDASH	Quick Disabilities of the Arm, Shoulder and Hand
TDAP	Thoracodorsal artery perforator
